# Cold plasma: A new technology to modify wheat flour functionality

**DOI:** 10.1016/j.foodchem.2016.01.113

**Published:** 2016-07-01

**Authors:** Niloufar Bahrami, Danny Bayliss, Gemma Chope, Simon Penson, Tania Perehinec, Ian D. Fisk

**Affiliations:** a The University of Nottingham, Division of Food Sciences, School of Bioscience, Sutton Bonington Campus, Loughborough LE12 5RD, UK; b Campden BRI, Station Road, Chipping Campden, Gloucestershire GL55 6LD, UK

**Keywords:** Wheat, Cold plasma, Food processing, Flour functionality

## Abstract

Atmospheric pressure cold plasma has the potential to modify biological chemistry and modulate physical surface properties. Wheat flour was treated by low levels of cold plasma (air, 15 V and 20 V) for 60 or 120 s. There was no change in the total aerobic bacterial count or total mould count as a result of treatment. Treatment did not impact the concentration of total non-starch lipids, or non-polar and glycolipids. However, treatment did reduce total free fatty acids and phospholipids and was dose dependent. Oxidation markers (hydroperoxide value and head space n-hexanal) increased with treatment time and voltage, which confirmed the acceleration of lipid oxidation. Total proteins were not significantly influenced by treatment although there was a trend towards higher molecular weight fractions which indicated protein oxidation and treated flour did produce a stronger dough. This study confirms the potential of cold plasma as a tool to modify flour functionality.

## Highlights

• Wheat flour was treated with low levels of cold plasma.
• Cold plasma treatment did not impact microflora.
• Cold plasma treatment altered the molecular weight distribution of wheat protein polymers.
• Cold plasma treatment oxidised free fatty acids and phospholipids.
• Wheat flour subjected to cold plasma treatment produced a stronger dough.

## 1 Introduction

A wide range of different wheat flour treatments (microwave, autoclave steaming, roasting, toasting and infrared heating) have previously been trialled to stabilise or modify the functionality of wheat flour (Kermasha et al., 1993, Sivri et al., 1992, Srivastava et al., 2007, Zwingelberg and Fretzdorff, 1996). Previous authors showed that different stabilisation techniques have different influences on wheat flour nutritional quality; for example, Demir and Elgün (2014) compared the effects of autoclave, microwave, infrared and UV-C on the bran fraction of wheat flour, and after re-blending, analysed the nutritional properties of whole wheat flour (crude protein, *in vitro* protein digestibility, phytic acid content, total and HCl extractable mineral concentrations, total phenolic compounds, antioxidant activity and total dietary fibre). All stabilization methods modified the nutritional characteristics of whole wheat bread, especially UV-C and infrared treatments, which had positive effects on proteins and antioxidant activity. Autoclave and microwave treatments increased the total extractable mineral and HCl extractable minerals and decreased extractable phytic acid content. Elgün, Demir, Bilgiçli, Türker, and Ertaş (2011) studied microwave heating and autoclaving and its effects on fine branny fractions and compared them with non-stabilised whole wheat flour. They showed that the storage stability of treated flour and bread increased by up to 90 days when compared with the untreated whole wheat flour.

In addition to stabilisation treatments, a range of other approaches have been trialled to improve wheat flour functionality via changes in the chemical composition; for example oxidising agents (chlorination, KIO_3_, KBrO_3_ and ascorbic acid) or oxidising enzymes (e.g. lipoxygenase) have been shown to modify wheat flour (Patrignani, Conforti, & Lupano, 2014) although they are not always approved for use in all food systems.

Ozone, due to its oxidising properties, has been demonstrated as an alternative to oxidising agents (Sandhu, Manthey, & Simsek, 2012) for the treatment of wheat flour and offers significant benefits; for example, it leaves no residues and when compared to chlorine, the oxidising influence of ozone is greater and the by-products produced from ozone treatment are proposed to be less harmful (Al-Dmoor, 2013). It should be stated that ozone received GRAS (Generally Recognised as Safe) status in the United States of America as a food processing aid following U.S. Food and Drug Administration (FDA) approval in 1997 (Cullen et al., 2009, Joshi et al., 2013). Recent studies have also shown that ozone can successfully modify wheat flour functionality (Chittrakorn, 2008, Misra et al., 2015, Sandhu et al., 2012).

Industrial ozone production usually involves the use of corona discharge in an oxygen rich gas. Dielectric barrier discharges are one of the most effective procedures to produce ozone (Alonso, Garcia, Calleja, Ribas, & Cardesin, 2005); it comprises of two electrodes with a large potential variance with a dielectric material between to inhibit the formation of arcs. The discharge from a dielectric barrier system results in a plasma containing a cascade of electrons, reactive ions and neutral species and with the emission of photons. Plasma at atmospheric pressure is usually characterised by a net gas temperature close to ambient, termed as cold plasma (Misra, Keener, Bourke, Mosnier, & Cullen, 2014). Research on cold plasma sources has grown strongly over the last few years, to some extent driven by possible medical ‘*in vivo*’ applications (Pompl et al., 2009).

Cold plasma has been shown to be a source of reactive oxygen species (ROS) including singlet oxygen, ozone and excited molecular nitrogen (Misra et al., 2014). This was found to be useful in the inactivation of a wide range of microorganisms including spoilage organisms and food borne pathogens (Brun et al., 2010, Niemira, 2012). In a recent study conducted by Misra et al. (2015) the effect of cold plasma treatment (60–70 kV, 5–10 min) on the rheological properties of soft and hard wheat flour was investigated, the results indicated an improvement in mixing time and dough strength (analysed by mixograph) after cold plasma treatment, attributed to oxidation of protein sulfhydryl groups and subsequent disulphide bond formation between cysteine moieties. The viscous modulus and elastic modulus of strong wheat flour increased with treatment time and applied voltage. In their study, changes that occurred in the rheological properties of the wheat flour were proposed to be associated with changes in the wheat flour protein.

Cold plasma has the potential to change the chemical composition of wheat flour through radical induced and ozone propagated oxidation and thereby change the functionality of the flour. However, the extent of the changes in the flour needs to be determined and controlled to enhance flour functionality. Therefore, this study was conducted to identify the effect of low doses of cold plasma treatment on wheat flour microbial load, chemical composition (protein and lipid) and dough rheology. A low treatment level was selected to minimise any secondary effects such as the formation of oxidation products that might be aroma active.

## 2 Materials and methods

### 2.1 Materials

Bread type wheat flour (*Triticum aestivum* L.) was prepared at Campden BRI (Gloucestershire, UK), and was matured for 2 weeks. The flour was then stored at −20 °C. All reagents were sourced from Sigma–Aldrich (Dorset, UK) and were of analytical grade.

### 2.2 Cold plasma treatment

Wheat flour samples (12 g) in petri dishes were treated for 60 s and 120 s using a continuous cold plasma prototype (the system was manufactured through collaboration with the University of Liverpool and Campden BRI and was funded by the EPSRC Impact Acceleration Account Scheme). A plasma discharge was generated using an operating frequency of 9 kHz with an applied power of 40 ± 1 W and 90 ± 1 W for DC power supply input voltages of 15 and 20 V respectively (this equated to applied powers of 0.19 W/cm^2^ and 0.43 W/cm^2^). The gas was the surrounding air on the day of treatment.

### 2.3 Aerobic mesophiles, thermophiles and associated spoilage fungi

For total aerobic bacteria count, wheat flour (3 g) was diluted tenfold using Maximum Recovery Diluent (MRD, Oxoid CM0733, Fisher Scientific, Loughborough, UK), and appropriate dilutions were spread plated in triplicate (0.1 mL of dilution) on Plate Count Agar (PCA, Oxoid CM0325, Fisher Scientific, Loughborough, UK). Plates were incubated at 30 °C for 24–48 h (mesophiles) and 42 °C for 24–72 h (thermophiles).

For the detection of moulds, wheat flour (3 g) was diluted ten-fold using MRD (Oxoid CM0733, Fisher Scientific, Loughborough, UK) and appropriate dilutions were spread plated in triplicate (1,0 mL of dilution) on Rose-Bengal Chloramphenicol agar (RBC, Oxoid SR0078, Fisher Scientific, Loughborough, UK). Plates were incubated aerobically at 25 °C for 3–5 days (limit of detection = 3 cfu/g). After incubation, colonies were enumerated. Results were expressed as log cfu/g of wheat flour.

### 2.4 Flour lipids

The Bligh and Dyer lipid extraction protocol (Bligh & Dyer, 1959) was used to extract non-starch lipids from cereal samples at ambient temperature. All samples were extracted three times by each extraction protocol, at ambient temperature as described by Bahrami et al. (2013). Isolates were evaporated under nitrogen, dissolved in chloroform (1 mL) and stored at −80 °C until required for lipid fractionation and the analysis of fatty acid methyl esters by gas-chromatography mass-spectrometry.

The most abundant fatty acids were palmitic (C16:0), stearic (C18:0), oleic (C18:1), linoleic (C18:2) and linolenic acid (C18:3), although others were identified (<5%). The total non-starch lipid was calculated and reported as the sum of the five major fatty acids (% w/w), to maximise comparability and minimise analytical variation that may be introduced by including low concentration lipid components. All analysis was carried out in triplicate.

#### 2.4.1 Lipid fractionation: solid phase extraction: SPE1

The isolated lipid fraction was then fractionated by a SPE process (SPE 1) into neutral lipids, glycolipids and phospholipids. Silica SPE columns (GracePure™ silica 1000 mg, 6 mL, Grace Discovery Science, UK) were conditioned using 5 mL hexane followed by 5 mL chloroform. The extracted lipid in chloroform (1 mL) was then added to the top of the silica SPE column and the drained solvent was collected and tested to ensure all lipids were loaded on the column. Chloroform:acetone (4:1, v/v) (10 mL) was added after the complete elution of the solvent; Neutral lipids were eluted at this step. Acetone:methanol (9:1, v/v) mixture solvent (15 mL) was added to elute glycolipids. Methanol (10 mL) was added to elute phospholipids. The flow rate of the SPE holder was adjusted to 0.7 mL/min by applying a vacuum manifold (Ohm & Chung, 1999). Prepared fractions were dried under nitrogen and dissolved in 1 mL chloroform to be analysed by GC–MS after direct derivatization by trimethylsulfonium hydroxide (TMSH).

#### 2.4.2 Lipid fractionation: solid phase extraction: SPE2

The neutral lipid fraction obtained from SPE 1 was then fractionated by a second SPE process (SPE 2) into non-polar lipids and free fatty acids. SPE columns (GracePure™ silica 1000 mg, 6 mL, Grace Discovery Science, UK) were pre-conditioned using 4 mL hexane. The neutral lipids prepared by SPE 1 in chloroform (1 mL) were loaded to the top of the silica SPE column. n-hexane:diethylether (8:2, v/v) (4 mL) was added, neutral lipids were eluted at this step (hydrocarbons, pigments, sterols, triglycerides and waxes). Free fatty acids (FFAs) were eluted by a mixture of n-hexane:diethylether (1:1, v/v) (4 mL). After the complete elution of solvent containing FFAs, methanol (4 mL) was added to wash out remaining polar lipids. After separating free fatty acids from the neutral lipids, the other two fractions obtained from SPE 2 were considered as non-polar lipids.

#### 2.4.3 Fatty acids

Crude and fractionated lipid extracts were evaporated to dryness under a nitrogen stream, dissolved in chloroform (1 mL) and stored at −80 °C until analyses. Lipid samples were thawed, combined with an internal standard (40 μg heptadecanoic acid in 20 μl chloroform) and derivatized to fatty acid methyl esters (FAME) with 200 μl of 0.25 M TMSH in methanol (10 min at ambient temperature) (Fisk, Gkatzionis, Lad, Dodd, & Gray, 2009). FAME samples (1 μl) were then separated by gas chromatography (Carlo Erba GC 8000, Milan, Italy; ZB-FFAP column, 30 m, 0.25 mm ID; oven temperature 120 °C held for 1 min then ramped at 5 °C/min to 125 °C, then ramped to 260 °C at 10 °C/min) and FAME were detected by mass spectrometry (DSQ II Thermoelectron, Austin, USA; positive ion mode, full scan from 50 to 385 *m*/*z*, scan rate 500 amu/s and scan time of 0.69 s, source temperature 200 °C). Compound identification was achieved by matching with database MS spectra (NIST/EPA/NIH Mass Spectral Library, Version 2.0d, NIST, Gaithersburg, USA), and comparing retention time and MS spectra with those of authentic FAME standards (Supelco, Bellafonte, USA). Quantification was achieved using the ratio of each target compound’s peak area relative to that of the internal standard.

#### 2.4.4 Lipid hydroperoxides (PV)

Wheat flour (0.5 g) was extracted using the Bligh and Dyer lipid extraction methodology with 3 replicate extraction steps. The extracted lipids were dried under nitrogen and dissolved in 1 mL chloroform. The extracted lipid in 1 mL chloroform (200 μl) was then analysed for hydroperoxide value (PV) using the method of Shantha and Decker (1994) as modified by Nuchi, McClements, and Decker (2001) and Fisk, White, Lad, and Gray (2008).

### 2.5 n-Hexanal

The abundance of n-hexanal was calculated in the sample headspace using solid phase micro-extraction gas chromatography mass spectroscopy (SPME-GCMS) (Beltran, Aguilera, & Gordon, 2005). Headspace abundance was determined using a CTS Analytics PAL system autosampler and a DSQ and Trace GC Ultra (Thermo Electron Corporation). Volatiles were quantified with authentic standards. Headspace volatiles were then separated by gas chromatography. GC equipment and setup parameters were: Carlo Erba GC 8000, Milan, Italy; ZB-wax column, 30 m, 0.25 mm ID, DVB/carboxen/PDMS stable flex, 10 mm, scale 36 mm, ID 24; pre incubation time 2 min, incubation temperature 45 °C, pre incubation agitator speed 500 rpm, agitator on time 0.05 min, agitator off time 0.02 min, vial penetration 22 mm, extraction time 15 min, inlet temperature 230 °C, split flow 20 mL/min, injection penetration 54 mm, description time 1.50 min, GC run time 2 min, oven temperature 40 °C held for 2 min then ramped at 8 °C/min to 48 °C, then ramped to 350 °C at 90 °C/min) and hexanal was detected by mass spectrometry (DSQ Thermo electron, Austin, USA; positive ion mode, selected ion monitoring 44, 56 and 72 and scan time of 0.23 s, source temperature 200 °C). Compound identification and quantification was achieved by matching with database MS spectra (NIST/EPA/NIH Mass Spectral Library, version 2.0d, NIST, Gaithersburg, USA), and comparing retention time and MS spectra with those of authentic standards (Supelco, Bellafonte, USA).

### 2.6 Wheat flour proteins

Size exclusion high-performance liquid chromatography (SE-HPLC) was used to determine the protein polymer size distribution of the white flour samples. The analysis was performed according to the Profilblé method developed jointly by ARVALIS and l’Institut National de Recherche Agronomique (Morel, Dehlon, Autran, Leygue, & Bar-L’Helgouac, 2000). Flour (160 mg) was combined with 20 mL 1% SDS (w/v) in 0.1 M phosphate buffer (pH 6.9) at 60 °C for 80 min to dissolve the soluble gluten forming proteins. The solution was sonicated (Misonix Microson XL2000, Qsonica, LLC, Newtown, CT, USA) to solubilise the polymeric gluten proteins, and then centrifuged (4200*g*) for 5 min. An aliquot of the supernatant was sealed in a HPLC vial ready for analysis. SE-HPLC analysis was conducted using a Jasco system (Great Dunmow, Essex, UK, UV-975 UV/VIS detector, PU-980-02 HPLC pump, LG-980-02 ternary gradient unit, DG-1580-53 3-line degasser, AS-950 sampler) operating with a TSK gel G 4000SW column (30 cm × 7.5 mm) and a TSK gel SK guard column (7.5 cm × 7.5 mm). The flow rate of the solvent, 0.1 M phosphate buffer containing 1.1% SDS, was 0.7 mL/min, and detection was performed at 214 nm.

The chromatograms were integrated using a combination of automated algorithms and manual rules developed as part of the Profilblé method. The resulting SE-HPLC trace has five identifiable peaks. Peak ratios were calculated as reported by Millar (2003). The overall performance of the columns on the day of analysis was determined by comparing the results for a protein standard (cytochrome C) with control values determined when the column was first used. Each combination of guard and analytical columns was characterised on first use by running 5 protein standards.

### 2.7 Functional properties

#### 2.7.1 Viscosity measurement (RVA)

A Rapid Visco Analyser (RVA) (Newport Scientific RVA A-4, Warriewood) was used to analyse the apparent viscosity of flour samples as a function of time, temperature and stirring. Flour (3 g) mixed with water (25 g) based on 12% moisture content (moisture analysis was conducted using a standard oven assay) and stirred at a constant speed (160 rpm) during the experiment. Temperature profiles used were: hold for 1 min at 50 °C, heat at 10 °C/min from 50 to 90 °C, hold for 2.5 min at 95oC, cool at 10 °C/min to 50 °C and hold for 1.5 min at 50 °C, pasting curves were performed in duplicate. Only the most extreme treatments were tested by RVA, Reomixer and by SE HPLC.

#### 2.7.2 Torque time analysis

A reomixer was selected for analysis of dough rheology as it was able to operate effectively with small sample sizes. The reomixer (Bohlin) consists of an instrumented 10 g planetary pin mixer, a microprocessor unit for signal conditions and software for data acquisition. The reomixer recorded the torque–time trace from the developing dough as described in the manual of methods for wheat and flour testing (Anonymous, 2010). Previous work at Campden BRI has developed a data reduction process using the principle of PCA analysis. This allows the samples to be plotted on a two-dimensional quality map. All samples were analysed in duplicate.

### 2.8 Experimental design and statistical analysis

All experiments were conducted with a fully balanced experimental design, with randomised analysis order and three sample replicates. Comparison between extraction methods was carried out by one-way analysis of variance and Tukey’s HSD *post hoc* test using IBM SPSS Statistics, version 19 (IBM Corp., Armonk, USA). Probability values lower than 0.05 were considered significant.

## 3 Results and discussion

### 3.1 Aerobic mesophiles, thermophiles and associated spoilage fungi

**Fig. 1:**
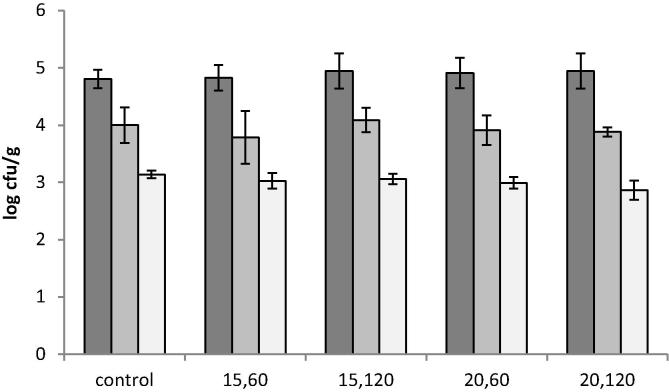
Total aerobic mesophylls, thermophiles and associated spoilage fungi after cold plasma treatment (control = no treatment). Total aerobic count at 30 °C (dark grey), at 42 °C (light gray) and mould count at 25 °C (white): 15, 60 (15 V, 60 s); 15, 120 (15 V, 120 s); 20, 60 (20 V, 60 s) and 20, 120 (20 V, 120 s).

It has been shown in previous studies that cold plasma treatment may reduce microbial viability (Brun et al., 2010, Choe et al., 2012, Fridman et al., 2007, Pompl et al., 2009, Venezia et al., 2008) and for flour, a reduction in bacterial and fungal contamination could be beneficial, therefore the flour was tested before and after treatment. No significant changes were found in total aerobic mesophiles (30 °C, 24/48 h) and thermophiles (thermophilic spore forming bacteria analysed at 42 °C, 24/72 h) after treatment (Fig. 1). A similar result was shown for the food associated spoilage fungi (mould) count (25 °C, 3–5 days) with no significant difference being found (Fig. 1). Ryu et al. (2013) reported that cold plasma efficiency for inactivating microorganisms varies depending on microbial species and the environment surrounding the microorganisms. For these flour samples the lack of evidence of microbial inactivation is attributed to the low moisture content (∼14%), low voltage and treatment duration.

### 3.2 Flour lipids

The impact of lipid oxidation on bread quality is poorly understood, but many authors (Chung et al., 1978, Cornell and Hoveling, 1998) have related factors such as bread volume to the oxidation of the natural lipids and proteins within wheat flours.

**Fig. 2:**
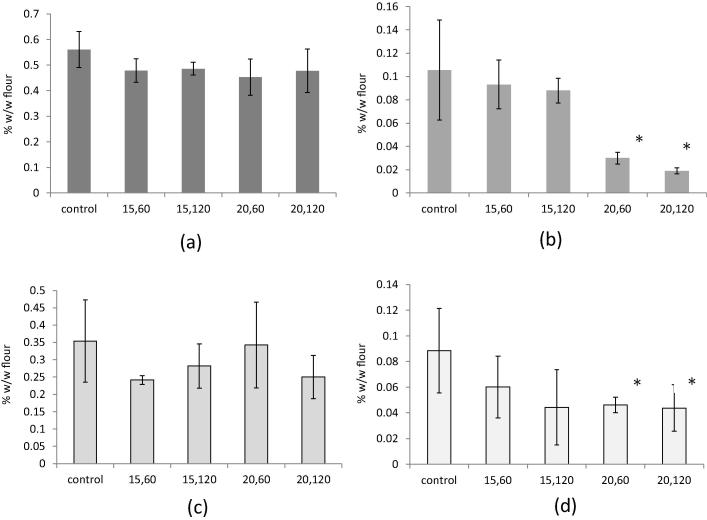
Different lipid classes (a) non-polar lipids, (b)FFAs, (c) glycolipids and (d) phospholipids fractionated from control and cold plasma treated wheat flour samples: 15, 60 (15 V, 60 s); 15, 120 (15 V, 120 s); 20, 60 (20 V, 60 s) and 20, 120 (20 V, 120 s), ^*^difference from control (*p* < 0.05).

When comparing the total extractable lipids of cold plasma treated wheat flour with untreated control flour no significant difference was found (data not shown). When the total extractable lipids were fractionated into different lipid classes (non-polar, FFA, glycolipid and phospholipid), no significant difference was found in the non-polar and glycolipids fractions (Fig. 2). However, the cold plasma treatment did have a significant effect on the free fatty acid and phospholipid complement of the wheat flour. Both of these species, known to be highly oxidatively labile, were significantly reduced by the higher voltage treatment (20 V) for 60 s or 120 s (Fig. 2). At the lower voltages there was no indication of lipid changes (no statistically significant change was observed between treated and untreated flour).

**Fig. 3:**
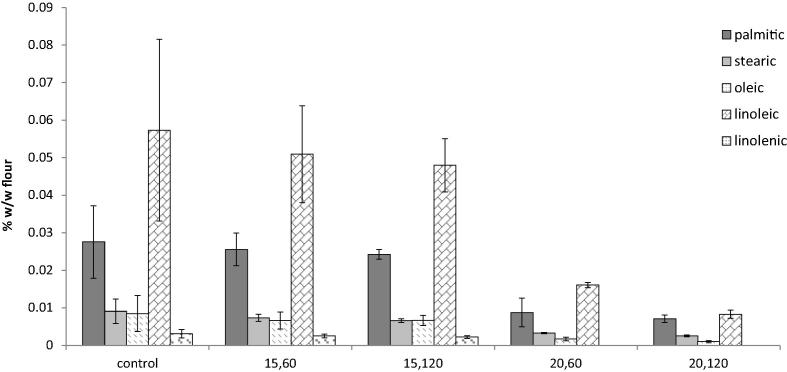
FFA composition of control and cold plasma treated wheat flour samples: 15, 60 (15 V, 60 s); 15, 120 (15 V, 120 s); 20, 60 (20 V, 60 s) and 20, 120 (20 V, 120 s).

The impact of cold plasma treatment on free fatty acids was due to a reduction in all major fatty acids (Fig. 3); this was more evident in the most oxidatively labile fatty acid, linolenic acid, which reduced by 100% at 20 V treatment when compared to the untreated control flour (Fig. 3).

**Fig. 4:**
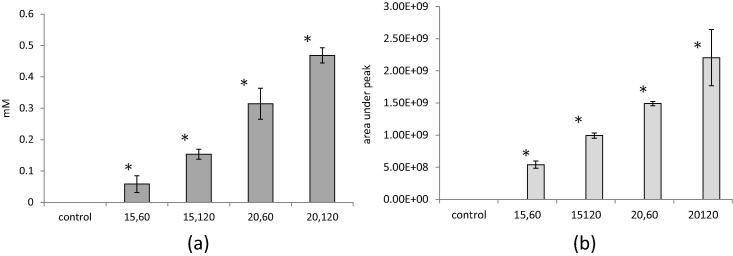
Lipid oxidation markers (a) PV and (b) n-hexanal of control and cold plasma treated wheat flour samples: 15, 60 (15 V, 60 s); 15, 120 (15 V, 120 s); 20, 60 (20 V, 60 s) and 20, 120 (20 V, 120 s). Control is close to zero and difference from control at *p* < 0.05 is indicated by ^∗^.

In order to determine the progression of lipid rancidity, primary (hydroperoxide value, PV) and secondary markers of lipid oxidation (n-hexanal) were determined. A similar trend was observed for both PV and n-hexanal abundance, in both cases there was a significant increase in the oxidation markers for all of the cold plasma treated wheat flour samples (Fig. 4). The development of both PV and hexanal abundance indicated a significant treatment effect on oxidation state.

### 3.3 Wheat flour proteins

In addition to the lipids being affected by the oxidative environment, the proteins could also be expected to be altered. Oxidation of wheat flour proteins is believed to directly impact a range of functional properties (Rosell et al., 2003, Thomasson et al., 1995). The oxidative changes in the protein may change their molecular weights and solubility (Rosell et al., 2003) and thus their interactions with water and their ability to form a gluten network. The impact of cold plasma treatment on the wheat flour total protein levels and the ratio of the different protein fractions is presented in Table 1A.

SE-HPLC was used to separate the total grain proteins (monomers and polymers) based on their size distribution, without reduction of disulphide bonds, with five peaks being recognised. The first peak to elute from the column is referred to as F1 and consists of HMW polymers enriched in HMW glutenin subunits (GS), while the F2 peak comprises LMW polymers and is enriched with LMW GS. The F3 and F4 peaks are comprised principally of ω-gliadins and α-, β-, and γ-gliadins, respectively, and the F5 peak comprises LMW proteins including albumins and globulins. The total area under the trace is a measure of the total protein content of the flour. No significant change was found in the total protein levels, but the flour subjected to the highest treatment (20 V for 120 s) had significant changes in the distribution of protein fractions and had a greater F1 fraction and lower F3, F4 and F5 fractions (as a fraction of total protein) (Table 1A). Overall this indicated a shift towards larger molecular weight (F1 + F2) proteins, this confirms the previous work by Misra et al. (2015) who showed changes in dough rheology proposed to be due to enhancement of disulphide bonds between glutenin subunits.

### 3.4 Wheat flour functional properties

The present study showed that the proportion of F1 + F2/F3 + F4 significantly increased in cold plasma treated flour and that there was enhanced oxidation of the lipids. To investigate if these changes had an impact on the functionality of the flour a series of functional tests were applied to the treated and non-treated flours.

**Fig. 5:**
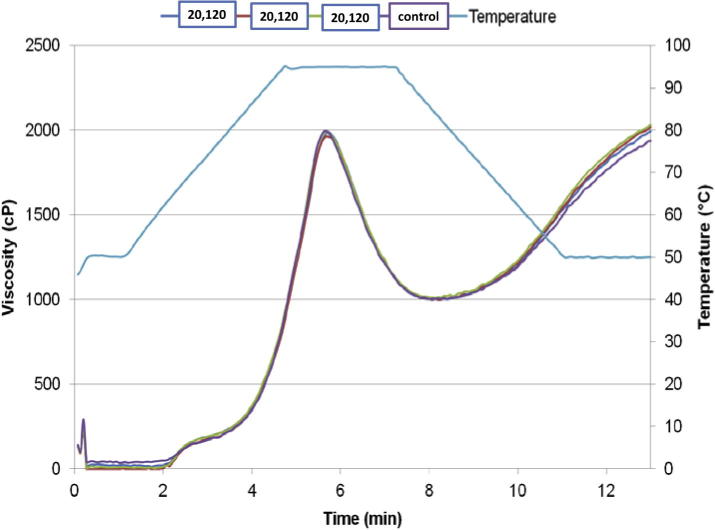
Pasting properties of wheat flour treated with cold plasma (20 V for 120 s – blue, red and green lines) and untreated control flour (purple line). (For interpretation of the references to colour in this figure legend, the reader is referred to the web version of this article.)

To test the general pasting characteristics the samples were assessed using an RVA. The wheat flour sample at the highest level of treatment (20 V for 120 s) showed no significant change in its pasting properties when compared to the untreated control flour (Fig. 5). The pasting curve is normally dominated by the starch fraction and if this was not altered, then the pasting curve could be expected to be unaffected. The quality of the protein may be assessed using a reomixer. No significant difference was found in PC1; however there was a significant impact of cold plasma treatment (20 V for 120 s) on PC2 (Table 1B) and this is indicative of the formation of a stronger dough. This would seem to indicate that at the higher energy levels the cold plasma treatment is sufficient to alter the proteins and lipids to the extent that they impact on the functionality of the flour. Other authors have implied that cold plasma can improve dough strength (Misra et al., 2015) through disulphide bond formation and impact loaf volume (Menkovska, Mangova, & Dimitrov, 2014), but have not previously presented these changes in functionality for industrially relevant low treatment levels in association with lipidic and protein characterisation.

## 4 Conclusion

In conclusion, a bread making quality wheat was milled and then successfully subjected to low level cold plasma treatment using applied powers of 0.19 W/cm^2^ and 0.43 W/cm^2^ for different time periods and the samples assessed for chemical and physical changes. Although cold plasma has been used previously to reduce the viability of microorganisms found in wheat samples, the low treatment levels used showed no reduction in the levels of aerobic mesophiles, thermophiles and associated spoilage fungi, this was presumably due to the low moisture levels and low treatment levels.

The treatment was effective in creating changes in the lipid components of the flour. Cold plasma treatment was found to result in a voltage and treatment time dependent reduction in non-starch FFAs and phospholipids and progression of oxidation was evidenced by a significant increase in PV and n-hexanal concentration at all levels of treatment, suggesting that cold plasma is resulting in an acceleration of flour oxidation.

A shift towards higher molecular weights in the protein profile, as analysed by SE-HPLC, was seen at the higher energy inputs which could be expected to increase the strength of dough made from the flour. This was indeed the case as small scale dough rheology tests (Reomixer) showed that treated wheat flour resulted in stronger dough.

Having shown that cold plasma could be effective in enhancing flour functionality it is recommended that consideration is given to optimise the cold plasma treatment and evaluate the process on a wide range of wheat flours, as this was not included in our study, this is especially true of whole wheat flours where acceleration of oxidation may be detrimental due to the higher lipid levels present.

Ultimately, this level of control of low levels of oxidation could be critical for enhancing functionality of wheat flour by promoting appropriate dough structure formation for new product development scientists and new ingredient designers.

## Figures and Tables

**Table 1:** (A) Total protein (area under the curve) and the proportion of five different protein fractions identified by SE-HPLC; F1 – HMW polymers, F2 – LMW polymers, F3 and F4 – monomers, F5 – LMW proteins for control and cold plasma treated (20 V for 120 s) wheat flour, ^*^significant difference at *p* < 0.05; ^ns^no significant difference. (B) Dough rheology of untreated control and cold plasma treated flour (20 V for 120 s) analysed by Reomixer. PC1 and PC2 are co-ordinates that represent data reduction of the torque–time data for each sample. Average and standard deviation of replication abbreviated as mean and SD.

	Total protein^ns^	F1^∗^	F2^ns^	F3^∗^	F4^∗^	F5^∗^
*(A)*						
Control	25.2 ± 0.27	13.3 ± 0.35	23.1 ± 0.27	8.40 ± 0.08	39.3 ± 0.11	16.0 ± 0.08
20, 120	25.5 ± 0.39	14.3 ± 0.29	23.4 ± 0.15	7.89 ± 0.12	38.5 ± 0.16	15.8 ± 0.02

	PC1		PC2	
	Mean	SD	Mean	SD
*(B)*				
Control	−5.96	0.676	3.05	0.384
20, 120	−5.82	0.406	−2.360	1.23
